# Observation of *Bothrops atrox* Snake Envenoming Blister Formation from Five Patients: Pathophysiological Insights

**DOI:** 10.3390/toxins13110800

**Published:** 2021-11-13

**Authors:** Sarah N. C. Gimenes, Jacqueline A. G. Sachett, Mônica Colombini, Luciana A. Freitas-de-Sousa, Hiochelson N. S. Ibiapina, Allyson G. Costa, Monique F. Santana, Jeong-Jin Park, Nicholas E. Sherman, Luiz C. L. Ferreira, Fan H. Wen, Wuelton M. Monteiro, Ana M. Moura-da-Silva, Jay W. Fox

**Affiliations:** 1 Laboratório de Imunopatologia, Instituto Butantan, São Paulo 05503-900, SP, Brazil; sarah.gimenes@univie.ac.at (S.N.C.G.); monica.colombini@butantan.gov.br (M.C.); luciana.sousa@butantan.gov.br (L.A.F.-d.-S.); 2 Escola Superior de Ciências da Saúde, Universidade do Estado do Amazonas, Manaus 69050-030, AM, Brazil; jsachett@uea.edu.br (J.A.G.S.); naajibe@gmail.com (H.N.S.I.); allyson.gui.costa@gmail.com (A.G.C.); monique.freire20@gmail.com (M.F.S.); wueltonmm@gmail.com (W.M.M.); 3 Departamento de Ensino e Pesquisa, Fundação de Dermatologia Alfredo da Matta, Manaus 69065-130, AM, Brazil; 4 Departamento de Ensino e Pesquisa, Fundação de Medicina Tropical Dr. Heitor Vieira Dourado, Manaus 69040-000, AM, Brazil; ferreira.luiz@gmail.com; 5 Departamento de Ensino e Pesquisa, Fundação de Hematologia e Hemoterapia do Amazonas, Manaus 69040-010, AM, Brazil; 6 School of Medicine, University of Virginia, Charlottesville, VA 22903, USA; jp2ht@virginia.edu (J.-J.P.); nes3f@virginia.edu (N.E.S.); 7 Núcleo de Produção de Soros, Instituto Butantan, São Paulo 05503-900, SP, Brazil; fan.hui@butantan.gov.br

**Keywords:** *Bothrops atrox*, blister, local damage, DAMPs, snake venom, antivenom, snakebite

## Abstract

In the Brazilian Amazon, *Bothrops atrox* snakebites are frequent, and patients develop tissue damage with blisters sometimes observed in the proximity of the wound. Antivenoms do not seem to impact blister formation, raising questions regarding the mechanisms underlying blister formation. Here, we launched a clinical and laboratory-based study including five patients who followed and were treated by the standard clinical protocols. Blister fluids were collected for proteomic analyses and molecular assessment of the presence of venom and antivenom. Although this was a small patient sample, there appeared to be a correlation between the time of blister appearance (shorter) and the amount of venom present in the serum (higher). Of particular interest was the biochemical identification of both venom and antivenom in all blister fluids. From the proteomic analysis of the blister fluids, all were observed to be a rich source of damage-associated molecular patterns (DAMPs), immunomodulators, and matrix metalloproteinase-9 (MMP-9), suggesting that the mechanisms by which blisters are formed includes the toxins very early in envenomation and continue even after antivenom treatment, due to the pro-inflammatory molecules generated by the toxins in the first moments after envenomings, indicating the need for local treatments with anti-inflammatory drugs plus toxin inhibitors to prevent the severity of the wounds.

## 1. Introduction

Snakebite envenoming (SBE) is classified as a neglected tropical disease that mainly affects rural areas in developing countries [1]. SBE victims display a wide array of symptoms and pathophysiology, largely depending on the type of snake that causes the wound, as well as intrinsic factors, such as the site of the bite, amount of venom injected, the health status of the victim, and the time elapsed before antivenom administration [2]. In Brazil, the northern states, which include the Amazon Forest, have the highest SBE prevalence. *Bothrops atrox* is the species responsible for the majority of SBE in the region, causing a significant detrimental economic and public health impact on the rural communities [3,4]. In this region, the typical local symptoms observed in *B. atrox* envenomation patients include edema, erythema, pain, and effects of tissue damage at the site of the bite, such as necrosis, inflammation and occasionally blistering in the proximity of the wound [5,6,7].

Blister formation following SBE, particularly in the case of *B. atrox,* occurs well after the bite [8,9]. Generally, blisters are related to a poor local prognosis as they increase the chance of infection and necrosis [10,11,12,13]. What is unknown is why there is such a significant delay in the formation of blisters following envenoming and why it appears antivenom treatment does not seem to prevent the appearance of blisters. This leads to a further question of what role venom components play in the pathophysiology of blister formation. It would seem unlikely that the venom components play a direct role in causing a blister given the delay in blister appearance, but perhaps it is possible they play an indirect role, whereby the actions of venom on the host ultimately lead to the production of agents or the activation of pathways that are known to produce blisters in other pathological conditions. 

Some studies have attributed blister formation to snake venom metalloproteinases (SVMPs) [14]. SVMPs represent a class of zinc-dependent enzymes with molecular masses ranging from 20 kDa to 110 kDa [15]. This family of venom proteinases has been shown to give rise to the signature of the local and systemic hemorrhage associated with viperid SBEs, as well as playing a role in the recruitment of an immune infiltrate and the local production of cytokines and chemokines [16,17,18]. These activities are primarily the result of the proteolytic abilities of the SVMPs to degrade a variety of extracellular matrix (ECM) components, including laminin, fibronectin, nidogen, and collagen, giving rise to both structural and functional contributions to the pathophysiology of SBE [19,20]. These toxins have also been implicated in inflammatory reactions associated with envenomation at the onset of local tissue damage [18]. Furthermore, it is relevant that viperid venom in general and its SVMP components, in particular, can produce a wound exudate rich in damage-associated molecular patterns (DAMPs), which contribute to chemokine and cytokine production and tissue permeability [12,13,21]. As such, it is possible that this is a contributing factor by which venom and/or SVMPs may contribute to blister formation. 

This report presents clinical and laboratory data derived from five *B. atrox*-envenomed patients focusing on blister production in these patients. These data are discussed to provide insight into blister formation in humans and how this may be important in other features of post-acute envenomation disabilities, and how this information may be considered for application in the clinical care of snakebite patients.

## 2. Results

### 2.1. Clinical Observations

The clinical descriptions of the patients included in this study are presented in Table 1. The severity of the envenomation was determined from the patient clinical data, which were primarily focused on the extent of local edema throughout the bitten limb and systemic manifestations of the patient [22]. Moderate envenomation is characterized by significant pain, apparent edema that goes beyond the envenomed anatomical site, and sometimes the presence of blistering. Severe envenoming is classified by the presence of intense pain and extensive edema, involving the entire envenomed limb, often accompanied by blistering, secondary infection, severe bleeding, hypotension, shock, and acute renal failure [22]. Based on these observations, three patients in the cohort (P1, P2, and P3) were clinically classified as suffering from “moderate” envenoming and two patients (P4 and P5) from “severe” envenoming. All patients, except one, were envenomed in the foot and there was a wide range of times recorded for the period of envenomation to hospitalization (2 h–11 h). Notably, all patients received antivenom at the time of admission. The times from envenoming until blister formation ranged from 63 h to 137 h. Surprisingly, a review of the clinical data did not show any correlation between the time of envenoming, hospitalization, antivenom administration, or envenoming severity with the time until the formation of blisters. However, this could simply be a reflection of the small sample size in this preliminary study.

The clinical data also provided insight into the systemic and local symptoms experienced in this patient group. Firstly, from a review of the systemic markers, an increased inflammatory state after envenomation was observed in all patients, as evidenced by increased levels of C-reactive protein (CRP). All patients experienced increased CRP levels from time 0 (immediately before antivenom treatment), which confirms that all patients had an acute inflammatory condition following envenoming (Table 1).

From the analysis of the local symptoms, all five patients reported intense pain on the day of hospital admission into the following day. All were noted to have the presence of moderate to severe edema during the time of hospitalization. Interestingly, in this particular patient cohort, blister formation was not evident until after a minimum of 58 h from the time of hospital admission (Table 1). Furthermore, at admission, all patients presented elevated levels of lactic dehydrogenase (LDH) (>190 UI/I), a marker indicative of tissue damage, suggesting local damage was well underway at the time of admission and subsequent antivenom administration. 

An important aspect common to the northern region of Brazil, and observed in some of these patients as well, is the long distances and difficulties in medical transport, hence the time from the accident to the patient being seen at the hospital is often longer than six hours [2]. The delays in patient care, along with the use of substances from traditional medicine and inappropriate practices, such as tourniquets, may aggravate the conditions at the bite site, leading to a high frequency of local complications resulting from the envenomings [9]. It is important to emphasize that one of our patients reported the use of tourniquets. Pardal and colleagues (2004) showed that tourniquet use is a common practice in *Bothrops* accidents [23]. Our data showed that the patient (Patient 4) who used a tourniquet before arrival at the hospital showed more severe inflammatory clinical parameters (Table 1). This observation highlights the importance of avoiding alternative methods of envenomation treatment, which may aggravate the patient’s clinical condition [22]. 

In general, it is considered that antivenom administration is relatively modest in its effectiveness at preventing local tissue damage from SBEs. The reason for the lower local efficacy of antivenom has been attributed to the low likelihood of the antivenom reaching the tissues in time to neutralize the critical venom components involved in local damage. In this study, all patients received antivenom therapy well after envenomation and all had delayed blister formation. This suggests that the mechanism through which blisters are formed, regardless of their delayed appearance, happens very early in envenomation. 

### 2.2. Laboratory Characterization for the Presence of Venom and Antivenom in Patient Serum and Blister Fluid

**Figure 1:**
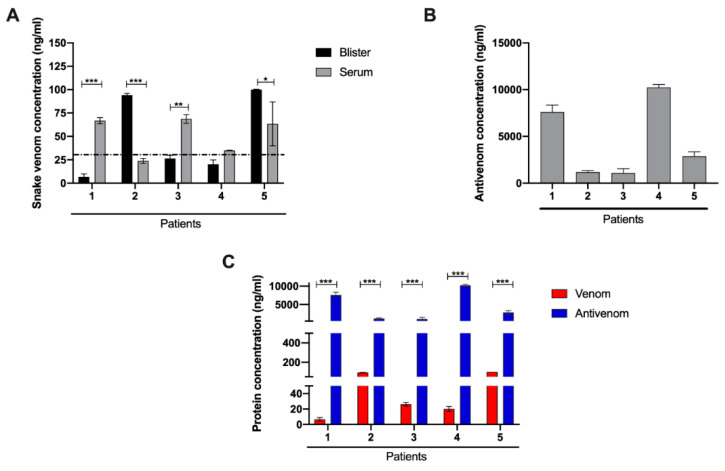
Quantitative analysis of snake venom and antivenom by enzyme-linked immunosorbent assay (ELISA). (**A**) Venom concentration in the serum at the time of patient admission and in the blister fluid. (**B**) Antivenom concentration in the blister fluid. (**C**) Comparative analysis of *B. atrox* venom and antivenom in the blister fluid. Results are expressed as the mean ± sd of three independent readings. *—*p*< 0.1; **—*p* < 0.05, ***—*p* < 0.01.

Previously, we have shown the presence of venom proteins in human blister fluid resulting from snakebites [12], and therefore we investigated whether venom proteins and antivenom were present in this cohort of patients’ blisters, as well as the relative amount of venom present in their serum. We used enzyme-linked immunosorbent assay (ELISA) to evaluate the venom protein concentration present in the serum of patients taken at the time of admission, before antivenom administration, and in their blisters that developed at much later times (Figure 1A). Not unexpectedly, the patients had varying venom concentrations in their serum, no doubt reflecting many different factors. Interestingly, patients 1, 3, and 5 who showed higher levels of serum concentration of venom were also the patients who had the shorter time intervals between envenomation and blister formation suggesting a higher venom amount injected at the time of the bite and thus potentially a more rapid and possibly higher production of the agents involved in subsequent blister formation. Another interesting observation from these data is that Patients 2 and 5 had higher venom concentrations in their blister fluid compared with their serum, suggesting that venom proteins were preferentially concentrated in the tissues adjacent to the wound.

Figure 1B shows the concentrations of antivenom measured in the contents of the blisters. The presence of antivenom was observed in blister fluids of all patients; however, the relative amounts of antivenom in the blister fluids were variable with Patients 1 and 4, being significantly higher than Patients 2, 3, and 5. Finally, we compared the protein concentrations of both venom and antivenom in the blister fluid (Figure 1C). In all cases, there appeared to be a higher protein concentration of antivenom compared with the venom in each blister, suggesting that the venom components are likely to be complexed with antivenom, with a significant amount of uncomplexed antivenom remaining in the blister. Hence, it is probable that most of the venom components found in the blister did not play a significant direct role in situ for blister formation, but acted to initiate a multi-factorial process well before the blister formation.

**Figure 2:**
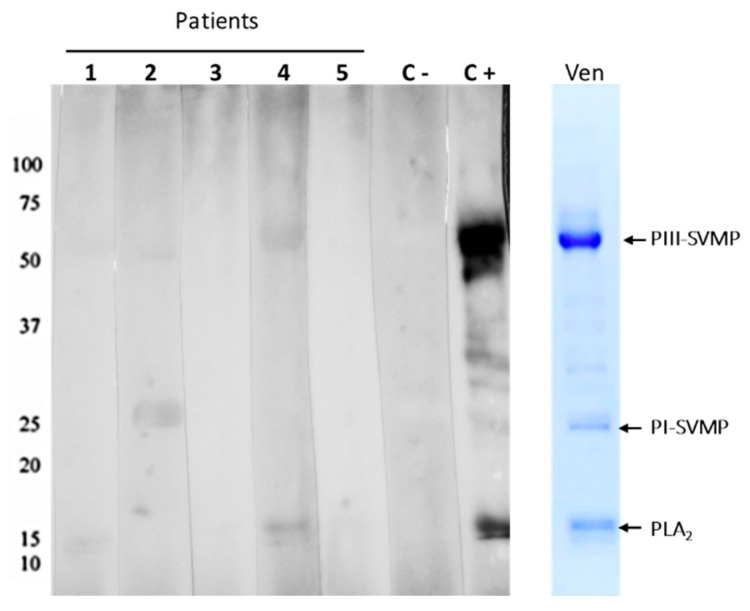
Recognition of *B. atrox* venom antigens by the antivenom present in the Blister fluids. *B. atrox* venom was electrophoresed under reducing conditions in 12% SDS-PAGE gels (Ven). Proteins contained in the gels were transferred to nitrocellulose membranes, which were incubated with the patients’ blister fluids (1–5), and the capacity of the antivenom present in the blister content to bind to venom proteins was detected by chemiluminescence using peroxidase-labeled anti-horse antibody and substrate. The images were captured after 15 s of membrane exposure. The positive control (C+) was the commercial antivenom similar to the one used to treat the patients, and the negative control (C−) was the plasma from a volunteer who never received antivenom as treatment. The numbers at the left indicate the migration of molecular mass markers and at the right, the bands corresponding to PIII-class snake venom metalloproteinases (SVMPs), PI-class SVMPs, and Phospholipases A_2_ (PLA_2_).

To determine whether uncomplexed antivenom was present in the blister fluids as well as to possibly get a sense of what venom components they could detect, we performed Western blots of the fluid against *B. atrox* venom. As seen in Figure 2, all patients’ fluids collected from the blisters contained antibodies able to react with *B. atrox* venom components. A closer examination of the Western blots suggested the presence of antibodies in Patients 1, 2, and 4, which could recognize higher molecular mass targets consistent with PIII-class SVMPs (~50 kDa). Interestingly, none of the blister fluids had antibodies that recognized the targets in the region of the PI-class SVMPs (~20 kDa). One potential explanation for this is that the antibody population in circulation represents the antibodies that remain after neutralizing venom components early in antivenom infusion. Thus, it may be that much of the PI-SVMP neutralizing antibodies were depleted from the antivenom before fluid filling these blisters, which appeared well after envenomation and antivenom treatment. However, we also have to consider that the *Bothrops* antivenom presents a higher reactivity to PIII-class SVMPs, recognizing preferentially epitopes located at the Disintegrin-like/Cysteine Rich Domains [24]. Regardless, it is fascinating that both venom, likely in complex with antivenom, and uncomplexed antivenom were detected in the blister fluid. The role this may play, if any, in blister formation will be discussed below.

### 2.3. Proteomic Characterization of Patient Blister Fluids

A total of 647 proteins were identified in the blister fluid from the five patients (Supplementary Table S1). Cluster analysis of these data did not provide any correlation to the clinical nor laboratory observations (data not shown). Proteins found in all of the blister fluids were compared to the control serum and included DAMPs, immunomodulators, complement, and ECM proteins (Table 2). Most of the complement proteins were of a lower abundance in the blister fluid than in the control serum, which is not surprising given that it is likely much of the complement system was activated and consumed by envenomation [25]. Interestingly, as observed in our previous studies [12,13,21], there was an increase in a variety of DAMPs in the fluids compared with the control serum, most notably the S100 protein family. In addition, several patients’ fluids showed an increase in ECM components, such as the collagens. Another observation was the increase in matrix metalloproteinase-9 (MMP-9) in four of the five patients’ blister fluids (Table 2). As MMP-9 has been implicated in blister formation, this was to be expected [26,27].

Following the molecular analysis of the endogenous factors, which likely contribute to local damage, we identified the presence of immunomodulators and DAMPs. These were generally observed to be at a higher abundance in the blister fluid than the normal serum (Table 2), reflecting the fact that DAMPs and immunomodulators are well-described markers associated with a pro-inflammatory effect. The presence of immunomodulators and DAMPs in blister fluid suggests that the local microenvironment could at some level be contributing to the blistering phenomenon.

Thus, from the analyses of the local effects, it suggests that blistering is a molecular process that may involve some snake venom toxins, from which SVMPs are good candidates. SVMPs are responsible for ECM protein degradation and the subsequent release of endogenous pro-inflammatory molecules, which increase vessel permeability and contribute to tissue damage and blister formation [21,28,29]. In this context, in the blister contents we found some products associated with ECM degradation, DAMPs, and immunomodulators triggered by snake venom toxins. Rucavado and colleagues (2016) investigated the presence of proinflammatory molecules in the exudate generated from the action of snake venoms in experimental models early after venom injection. Proteomics studies showed the presence of cytokines and DAMPs released after 1 h of snake venom inoculation. In addition, the injection of these same exudates into mice skin increased vascular permeability. These findings suggest that DAMPs and cytokines present in the exudate may interfere with hemostasis through inflammatory pathways, such as Toll-like receptors (TLRs) [21].

The molecular mechanism for tissue damage and blister formation in SBEs by activating the cascade of immunomodulators and TLRs can also be related to endogenous proteinases (MMPs). Endogenous proteinases can be released by infiltrating inflammatory cells triggering proteolytic and inflammatory cascades, which indicates a crucial role of a metalloprotease in wounds [27,30]. In bullous pemphigoid disease, an increasing perivascular inflammatory infiltration and activity of endogenous metalloproteinases, especially MMP-9 enhancing blisters, has been observed [26,27]. The presence of MMP-9 in the blister content from our patients suggests a combined action of endogenous and exogenous proteinases in pathophysiological events. MMPs can be up-regulated by the activation of Toll-like receptor pathways (TLR-4 and TLR-2) [31]. This leads to the speculation that MMP-9 in the blister may act on local ECM proteins and contribute to the blistering effect.

## 3. Discussion

In general, there is a paucity of studies on snake venom-induced blistering, although when observed in patients they can be quite dramatic, even though they do not significantly contribute to venom morbidity. In one study using an animal model of toxin-induced blister formation, a PI SVMP, BaP1, from *Bothrops asper* was shown to cause blister formation within an hour of injection of 80 µg of the toxin into mouse muscle [14]. In the wound exudate, endogenous MMPs and the toxin were identified along with several extracellular matrix fragments. This suggested to those authors that the SVMP and the endogenous MMPs likely were involved in blister formation via proteolytic degradation of matrix proteins involved in epidermal/dermal association. Another investigation of blister formation utilizing the same toxin, but in a mouse ear model, also produced a rapid formation of blisters [32]. The authors attributed blister formation to the direct proteolytic action of the SVMP on known substrates at the dermal–epidermal junction, such as collagen type IV and laminin. In spite of the use of these elegant models, it is difficult to expect that they fully reflect the situation of blister formation in envenomed humans. In the mouse studies, the authors used a relatively high concentration of the toxin, which could be why blisters formed so rapidly in those models and thus could be attributed to a direct effect of the toxin on key proteins involved with dermal/epidermal association. Furthermore, mouse skin is quite different from human skin, primarily in that human skin is significantly thicker and firm, whereas mouse skin is thinner and loose, being comprised of only two or three cell layers in the epidermis compared to five to ten in humans [33]. Therefore, it seems unlikely that the mechanism of blister formation by the direct action of SVMPs at the dermal–epidermal junction is the same in humans, and thus this warrants different considerations.

In this study, we identified the presence of immunomodulators and DAMPs in blister fluid. These were generally observed to be at a higher abundance in the blister fluid in comparison with normal serum, reflecting the fact that DAMPs and immunomodulators are typically associated with a pro-inflammatory effect, contributing, at some level, to blistering phenomenon. Another important observation was that both antivenom and venom reach and remain in the envenomation region for a long period. However, the presence of antivenom in the blister did not prevent local tissue damage or blister formation. These observations highlight the immediacy of venom action at the site of envenomation activating endogenous pathways that promote tissue damage even after antivenom administration. In this study, it was evident that antivenom reaches the injured limb, and the activation of such endogenous pro-inflammatory pathways explains the general considerations that antivenom administration is relatively modest in its effectiveness in preventing local tissue damage in patients. Blister formation could be an independent process of the local protective effect of antivenom.

In this context, our results suggest that the pathophysiology of blister formation is more likely related to the generation of proinflammatory molecules (DAMPs and immunomodulators) by the toxins before antivenom administration. Although blisters do not play a significant role in morbidity, our investigation also illustrates how the initial action of the venom can contribute to pathophysiology that occurs at a relatively long period after envenoming, regardless of treatment with antivenom. The mechanism by which blisters are formed may be extended to other effects, leading to tissue damage in these patients, which may also be induced before antivenom administration. Finally, as has been long recognized, there remains an urgent need for therapeutic approaches that can rapidly be deployed at the envenomation site to attenuate the toxic activities that occur virtually immediately and have long-lasting effects, both locally and systemically. In this regard, our data strongly support the need for local treatments with anti-inflammatory drugs plus toxin inhibitors to prevent the severity of the wounds.

## 4. Materials and Methods

### 4.1. Ethical Statement and Clinical Data Collection

Patients included in the study (5) were hospitalized due to snakebite, at the Fundação de Medicina Tropical Dr. Heitor Vieira Dourado (FMT-HVD), a reference hospital for snakebite treatment in Manaus, State of Amazonas, Brazil. Eligible patients were those diagnosed with clinical signs of envenomation by *Bothrops* snakes, which developed blisterings at the bitten limb during the time of hospital treatment. This study was approved by the Ethics Review Board of FMT-HVD (approval number CAAE 19380913.6.0000.5016/2013). All participants signed a consent form after an explanation of the study aims. Upon admission, epidemiological and clinical information were collected using a standardized questionnaire. Envenomation was classified as mild, moderate, or severe, according to the Brazilian Ministry of Health guidelines [22]. The presence of pain, local bleeding, ecchymosis, necrosis, and systemic bleeding were also recorded. Patients were treated according to the Brazilian Ministry of Health protocols [22]. To collect the blister fluids, the area was cleaned using 2% chlorhexidine, and the fluid was collected using a sterile needle (13 × 4.5 mm) and a 1mL syringe. Subsequently, blister fluid was aliquoted and transferred to a sterile microtube and frozen at −80 °C until analysis.

### 4.2. ELISA Quantification of Antivenom and Venom in Blister Contents

For antivenom detection, a 96-well plate was coated for 18 h at room temperature with 10 µg/mL of *B. atrox* venom in PBS (100 µL/well). The plates were then washed with PBS containing 0.05% TWEEN 20 solution and then blocked for 2 h at 37 °C with 300 μL/well of phosphate-buffered saline (PBS) containing 2% bovine serum albumin (BSA). After blocking, the plates were washed and incubated for 2 h at 37 °C with the blister fluid 1000 times diluted in PBS containing 1% BSA and 0.05% TWEEN 20 or different dilutions of the standard curve, prepared with a known concentration of IgG (Fab’_2_ fragment) isolated from the same antivenom used for patient treatment. After a new washing step, peroxidase-labeled anti-horse IgG antibodies (SIGMA) were placed in each well, 2000 times diluted, and incubated at 37 °C for one hour. Antigen/antibody binding was detected by the addition of ortho-phenylenediamine. The antivenom concentration was calculated by linear regression of the standard curve. The same methodology was used for the detection of venom in the blister fluids, in which the plates were coated with Bothrops antivenom in a PBS buffer. After washing, the plates were incubated for 4 h at 37 °C with blister fluid (diluted 1:5 in PBS containing 1% BSA and 0.05% TWEEN 20) and the standard curve was prepared with *B. atrox* venom at known concentrations. The reaction was detected by rabbit serum anti-*B. atrox* venom followed by the addition of peroxidase labelled sheep anti-rabbit IgG (SIGMA) and ortho-phenylenediamine. The plates were read using a wavelength of 492 nm, and the venom concentration was calculated by linear regression of the standard curve. Experiments were carried out in triplicate and the results were expressed as mean ± S.E.M.

### 4.3. Western Blot of Antivenom in Blister Contents

The reactivity of blister contents with venom proteins was evaluated by Western blot. Briefly, *B. atrox* venom was electrophoresed under reducing conditions in 12% dodecyl sulfate-polyacrylamide gel electrophoresis (SDS-PAGE) gels. Proteins contained in the gels were transferred to nitrocellulose membranes, which were blocked for 2 h at 37 °C with 500 μL of PBS containing 2% BSA (SIGMA-USA). Then, each membrane was incubated overnight at 4 °C with blister fluids from each patient, diluted at 1:2 in PBS containing 1% BSA. Following incubation, the membranes were washed with PBS containing 0.05% TWEEN. The capacity of the antivenom present in the blister content to bind to snake venom proteins immobilized on the nitrocellulose membranes was detected by chemiluminescence using peroxidase-labeled anti-horse antibody and ^®^Super Signal West Pico substrate from Thermo Scientific (Waltham, MA, USA). The images were captured after 15 s of membrane exposure. The positive control was Butantan commercial antivenom, similar to the one used to treat the patients, and the negative control was the plasma from a volunteer who never received antivenom as treatment.

### 4.4. Proteomic Analyses

The proteins (~10 ug) contained in the patient blisters and the serum of normal volunteers were submitted to reduction with 10 mM dithiothreitol (DTT) in 0.1 M ammonium bicarbonate and alkylation with 50 mM iodoacetamide in 0.1 M ammonium bicarbonate (both room temperature for 0.5 h). The samples were then digested overnight at 37 °C with 1 µg trypsin in 50 mM ammonium bicarbonate. The samples were acidified with acetic acid to stop digestion and were then spun down. The supernatant was evaporated to 20 µL for liquid chromatography–mass spectrometry (LC–MS) analysis.

The LC–MS system utilized for proteomic analyses was a Thermo Electron Q Exactive HF mass spectrometer system with an Easy Spray ion source connected to a Thermo 75 µm × 15 cm C18 Easy Spray column (through pre-column). Samples of 1 µg were injected and the peptides eluted from the column by an acetonitrile/0.1 M acetic acid gradient at a flow rate of 0.3 µL/min over 2.0 h. The nanospray ion source was operated at 1.9 kV. The analysis produces approximately 25,000 MS/MS spectra of ions ranging in abundance over several orders of magnitude. The data were then analyzed by database searching using the Sequest search algorithm against Uniprot Human. An analysis of the spectra generated was performed using carbamidomethylation on cysteine as a fixed modification, with oxidation of methionine as a variable modification. For the analysis of the results and validation of peptide and protein identifications, data obtained were exported to Scaffold (version 4.3.2, Proteome Software Inc., Portland, OR, USA).

## Figures and Tables

**Table 1:** Clinical data of patients.

Clinical Data/Patient		Patient 1	Patient 2	Patient 3	Patient 4	Patient 5
Envenomation severity		Moderate	Moderate	Moderate	Severe	Severe
Gender		Male	Female	Male	Male	Male
Bite site		Foot	Foot	Foot	Leg	Foot
Tourniquet use		No	No	No	Yes	No
Time until hospital admission		5 h	2 h	6 h	11 h	4 h
Time from hospital admission to blister appearance		58 h	135 h	77 h	111 h	80 h
Time from envenomation to blister appearance		63 h	137 h	83 h	122 h	84 h
Blister site *		Foot	Foot	Foot	Leg	Foot
Pain	Time 0	10	8	7	10	10
	Time 24	4	7	5	0	5
(0 to 10 scale) **	Time 48	0	5	4	0	0
	Time 72	0	5	3	0	7
	Time 144	0	4	2	8	0
Edema ***	Time 0	Moderate	Moderate	Mild	Moderate	Moderate
	Time 24	Moderate	Moderate	Moderate	Moderate	Severe
	Time 48	Moderate	Moderate	Moderate	Moderate	Severe
	Time 72	Moderate	Moderate	Moderate	Moderate	Moderate
	Time 144	Moderate	Mild	Mild	Moderate	Moderate
Ecchymosis		No	After 48 h	No	No	No
Necrosis		No	No	No	No	No
Infection		No	Yes	Yes	Yes	Yes
Lactic Dehydrogenase ^#^	Time 0	464	234	715	935	264
	Time 24	335	221	501	305	255
	Time 48	308	115	281	286	344
	Time 72	313	283	302	328	255
	Time 144	285	263	289	230	289
C-reactive protein ^##^	Time 0	6,5	6,5	48	96	6,5
	Time 24	96	96	48	96	48
	Time 48	96	96	192	24	48
	Time 72	24	96	96	48	96
	Time 144	48	96	96	12	48

* In all patients, blisters were hemorrhagic and appeared in the perilesional area, next to the bite. ** Pain (0–10) scale of 0 to 10 with 10 as highest; *** Edema was classified according to its extension, in segments as recommended by the Brazilian Ministry of Health [22]: mild = 1 to 2 segments, moderate = 3 to 4 segments, and severe = 5 affected segments. ^#^ Lactic Dehydrogenase normal value 190 UI/l; ^##^ C-reactive protein normal value 0.8 mg/dL.

**Table 2:** Most abundant extracellular matrix proteins, immunomodulators, and damage-associated molecular patterns (DAMPs) identified in blister fluids.

Identified Proteins	Ac. Number	Patient 1	Patient 2	Patient 3	Patient 4	Patient 5	Control Serum
Complement C3	P01024	106	128	69	264	101	122
Complement factor B	P00751	2	4	4	4	5	56
Complement C4-B	P0C0L5	0	0	3	28	2	37
Complement component C7	P10643	0	0	0	0	1	25
Complement component C9	P02748	0	0	0	0	1	21
Complement factor H	Q03591	0	0	5	0	5	20
Complement factor I	P05156	0	0	1	0	0	16
Complement C1s	P09871	0	1	2	3	1	16
Complement component C8	P07357	0	0	2	0	1	15
Complement C1r	Q9NZP8	1	0	0	4	0	13
Complement factor H-related protein 1	Q03591	0	0	0	0	1	2
Complement C2	Q8SQ75	4	5	0	22	2	0
Fibrinogen alpha chain	P02671	32	37	11	30	14	13
Fibrinogen gamma chain	P02679	1	3	9	7	12	0
Fibrinogen beta chain	P02675	0	2	12	6	11	0
Serum amyloid A-4	P35542	0	0	2	1	2	9
Proteoglycan 4	Q92954	0	2	0	0	0	2
Heat shock 70 kDa protein 1B	P0DMV9	6	25	18	6	37	0
Heat shock protein HSP 90-alpha	P07900	0	0	4	0	3	0
Heat shock protein HSP 90-beta	P08238	0	0	1	0	2	0
Basement membrane-specific heparan sulfate proteoglycan core protein	Q05793	0	0	0	1	0	0
Putative histone H2B type 2-C	Q6DN03	0	0	4	2	0	0
Histone H4	P35059	0	8	30	15	0	0
Myosin regulatory light chain sqh	P40423	0	0	1	0	1	0
Myosin-9	P35579	0	6	50	6	84	0
Protein S100-A4	P26447	11	10	6	4	4	0
Protein S100-A6	P06703	3	4	2	0	5	0
Protein S100-A8	P05109	2	5	6	8	23	0
Protein S100-A9	P06702	0	10	19	11	44	0
Protein S100-A12	P80511	0	6	6	6	17	0
Protein S100-A11	P31949	2	3	7	4	8	0
Protein S100-P	P25815	3	5	3	3	8	0
Annexin A1	P04083	0	0	0	1	4	0
Annexin A3	P12429	0	5	0	4	56	0
Annexin A5	Q5R1W0	0	0	0	0	19	0
Annexin A6	P08133	0	0	0	0	4	0
Alpha-2-HS-glycoprotein	P02765	30	15	5	25	3	39
Vitronectin	P04004	2	6	2	4	6	32
Fibronectin	P04937	0	1	4	0	2	17
Lumican	P51884	7	0	1	14	0	10
Proteoglycan 4	Q92954	0	2	0	0	0	2
EGF-containing fibulin-like extracellular matrix protein 1	O35568	0	0	1	1	0	2
Collagen alpha-1(III) chain	P02461	0	0	0	1	0	0
Collagen alpha-1(I) chain	P02452	1	0	2	12	3	0
Collagen alpha-1(VI) chain	P12109	0	0	0	4	0	0
Collagen alpha-1(XXVII) chain	Q5QNQ9	0	0	1	0	0	0
Collagen alpha-3(VI) chain	P12111	0	0	1	0	0	0
Matrix metalloproteinase-9	P14780	0	1	6	1	13	0
Olfactomedin-4	Q6UX06	0	0	0	4	21	0

The numbers in each cell are the abundance, according to the normalized spectra count. The red boxes represent values twice higher; blue boxes represent values twice lower; white boxes represent values without significant difference compared to the normal human serum.

## Supplementary Materials

The following are available online at https://www.mdpi.com/article/10.3390/toxins13110800/s1, Supplementary Table S1: Protein identification in blister fluids of *B. atrox* snakebite patients and serum of control volunteers by LC-MS.
